# Editorial Notes

**Published:** 1931

**Authors:** 


### Editorial Notes

**Colston Medical Research Fellowships.**

THE Council of the Colston Research Society have decided to use the Medical Research Fund collected by Mr. Falk for founding one or more Colston Medical Research Fellowships of not less than £100 per annum. The regulations governing these Fellowships are as follows, viz.:—

1. That the Fellowships be awarded by the Council of the Colston Research Society.

2. That they shall be Research Fellowships primarily in clinical subjects, but other medical subjects are not excluded.

3. That they be open to any member of the medical profession in Bristol, preference being given to graduates or members of the teaching staff of the University. Holders must possess a medical qualification, and their proposed work shall have been approved by the Council of the Society.

4. That the work shall be carried out in the University of Bristol or in any of the clinical institutions associated with the University.

5. That the holder be required to present a report at the end of the year on his work.

6. That the Fellowships be renewable yearly at the discretion of the Council of the Society.

7. That the Fellowships need not necessarily be awarded in any given year.

8. Applications should be made to the Hon. Secretary of the Society, 12 Small Street, Bristol, on or before 30th April of each year.

**Medical Dinner.**

THE Annual Medical Dinner was held on Thursday, 10th December, at the Clifton Zoological Gardens, under the Chairmanship of Dr. Charles Corfield. It was attended by nearly one hundred and fifty guests, the majority of whom were past or present Bristol students, thereby giving the Dinner the character of a reunion for the Bristol Medical School. This was the idea with which the Dinner was originally founded in 1882.

The guest of the evening was Professor D. C.

Rayner, who holds the Chair of Obstetrics in the University of Bristol, and is an alumnus of the Bristol Medical School.

The speeches were admirably short and to the point, whilst the banqueting-room and the catering arrangements gave the greatest satisfaction. In the course of the evening the Dean (Professor Fawcett) announced that a Committee had been formed to arrange the Centenary Celebrations of the Bristol Medical School in 1933.

**Edward Jenner's Books for the Medical Library.**

DR. ACHESON of Berkeley recently presented to our Library four interesting books which had been the property of Jenner, three of them still showing his signature. The great collection of Jenner's relics which was on exhibition here some years ago having gone to London and the United States permanently, we are fortunate to get these few books from his library.

The first we take up is *Observations on Scrophulous Affections*, by Robert Hamilton, M.D., published in 1791, an abstract of which had been read by Lettsom before the Medical Society of London and praised by it. The author at that time was a rising man, who had spent some years in the Army, in successful private practice, but unfortunately he became blind and remained so for thirty years.

Next we find a presentation copy of J. Mason Cox's *Practical Observations on Insanity*, dated 1806, the second edition. Cox was a Bristol man who graduated at Leyden, practised like so many others in Unity Street, and carried on an asylum at Fishponds.

Another volume presented by the author to Jenner is Sir J. C. Hippisley's *Speech on the Petition of the Roman Catholics in Ireland* in 1810, with many documents on the subject. Hippisley, then living at Stoneaston, and member for Sudbury, belonged to a famous family connected with Bristol, and had himself done a good deal for the city. As a diplomat he had successfully negotiated the marriage of the Princess Royal of England with the Duke of Wurtemberg, and was made a baronet as a reward. He had also obtained relief from King George III for the poverty-stricken Cardinal York, the last of the Stuarts.

Finally, there is a fine volume with many illustrations, *A Complete Treatise of Electricity*, by Tiberius Cavallo, a naturalized Italian, Fellow of the Royal Society, and one of the ablest scientists of the time. Cavallo seems to have been especially noted for the delicacy and accuracy of his measurements in electricity and physics.

